# Assembly and analysis of 100 full MHC haplotypes from the Danish population

**DOI:** 10.1101/gr.218891.116

**Published:** 2017-09

**Authors:** Jacob M. Jensen, Palle Villesen, Rune M. Friborg, The Danish Pan-Genome Consortium, Thomas Mailund, Søren Besenbacher, Mikkel H. Schierup

**Affiliations:** 1 Bioinformatics Research Centre, Aarhus University, 8000 Aarhus C., Denmark;; 2 Department of Clinical Medicine, Aarhus University, 8200 Aarhus N., Denmark;; 3 Department of Molecular Medicine, Aarhus University Hospital, Skejby, 8200 Aarhus N., Denmark;; 4 Department of Bioscience, Aarhus University, 8000 Aarhus C., Denmark; 6 Bioinformatics Centre, Department of Biology, University of Copenhagen, 2200 Copenhagen N, Denmark; 7 Bioinformatics Research Centre, Aarhus University, 8000 Aarhus C, Denmark; 8 iSEQ, Centre for Integrative Sequencing, Aarhus University, 8000 Aarhus C, Denmark; 9 DTU Bioinformatics, Department of Bio and Health Informatics, Technical University of Denmark, Kemitorvet, 2800 Kongens Lyngby, Denmark; 10 BGI-Europe, Ole Maaløes Vej 3, 2200 Copenhagen N, Denmark; 11 Department of Clinical Medicine, Aarhus University, 8000 Aarhus C, Denmark; 12 Novo Nordisk Foundation Center for Basic Metabolic Research, Section of Metabolic Genetics, University of Copenhagen, 2100 Copenhagen Ø, Denmark; 13 Department of Biomedicine, Aarhus University, 8000 Aarhus C, Denmark; 14 The Lundbeck Foundation Initiative for Integrative Psychiatric Research, iPSYCH, 8000 Aarhus, Denmark; 15 BGI-Shenzhen, Shenzhen 518083, China; 16 School of Bioscience and Biotechnology, South China University of Technology, Guangzhou 510006, China; 17 Laboratory of Genomics and Molecular Biomedicine, Department of Biology, University of Copenhagen, 2100 Copenhagen Ø, Denmark; 18 Department of Psychology, University of Oslo, 0317 Oslo, Norway; 19 NORMENT, KG Jebsen Centre for Psychosis Research, Department of Clinical Science, University of Bergen, Bergen, 5021, Norway; 20 Dr. E. Martens Research Group of Biological Psychiatry, Center for Medical Genetics and Molecular Medicine, Haukeland University Hospital, Bergen, 5021, Norway; 21 Department of Medical Epidemiology and Biostatistics, Karolinska Institutet, Stockholm, 17177, Sweden; 22 Department of Genetics, University of North Carolina, Chapel Hill, NC 27599-7264, USA; 23 Department of Clinical Epidemiology (formerly Institute of Preventive Medicine), Bispebjerg and Frederiksberg Hospital, The Capital Region, Copenhagen, 2000 Frederiksberg, Denmark; 24 Department of Public Health, Faculty of Health and Medical Sciences, University of Copenhagen, 2200 Copenhagen, Denmark; 25 Department of Cellular and Molecular Medicine, University of Copenhagen, 2200 Copenhagen N, Denmark; 26 Novo Nordisk Foundation Center for Protein Research, Faculty of Health and Medical Sciences, University of Copenhagen, 2200 Copenhagen N, Denmark; 27 Department of Bioscience, Aarhus University, 8000 Aarhus C, Denmark

## Abstract

Genes in the major histocompatibility complex (MHC, also known as HLA) play a critical role in the immune response and variation within the extended 4-Mb region shows association with major risks of many diseases. Yet, deciphering the underlying causes of these associations is difficult because the MHC is the most polymorphic region of the genome with a complex linkage disequilibrium structure. Here, we reconstruct full MHC haplotypes from de novo assembled trios without relying on a reference genome and perform evolutionary analyses. We report 100 full MHC haplotypes and call a large set of structural variants in the regions for future use in imputation with GWAS data. We also present the first complete analysis of the recombination landscape in the entire region and show how balancing selection at classical genes have linked effects on the frequency of variants throughout the region.

The major histocompatibility complex covers 4 Mb on Chromosome 6 and is the most polymorphic part of the human genome. Most of approximately 200 genes in the region are directly involved with the immune system. The high diversity is thought to be driven by balancing selection acting on several individual genes combined with an overall small recombination rate in the MHC (DeGiorgio et al. 2014). Genome-wide association studies have revealed the MHC to be the most important region in the human genome for disease associations, in particular for autoimmune diseases (Trowsdale and Knight 2013; Zhou et al. 2016).

The very high diversity and wide-ranging linkage disequilibrium (LD) makes it difficult to disentangle selective forces and to accurately pinpoint the variants responsible for disease associations. Many regions are too variable for reliable identification of variants from mapping of short reads to the human reference genome. LD causes multiple nearby variants to provide the same statistical evidence of association hampering the identification of causal variants. In addition to the human genome reference MHC haplotype, seven other haplotypes have been sequenced (Horton et al. 2008), although six of these are incomplete, and exploiting these significantly improves mapping performance (Dilthey et al. 2015, 2016). There is a strong need for obtaining a larger number of full MHC haplotypes, which requires de novo assembly of the haplotypes without the use of a reference genome. Long-read technology and refined capture methods are potentially very powerful (Chaisson et al. 2015; English et al. 2015; Selvaraj et al. 2015), but these approaches are still prohibitively expensive at a large scale.

The Danish Pan-Genome project (Maretty et al. 2017) was designed to perform individual de novo assembly of 50 parent–child trios sequenced to high depth with multiple insert size libraries. We use data from 25 of these trios to reconstruct and analyze the four parental MHC haplotypes in each trio (100 haplotypes in total). Our approach combines the de novo assemblies with transmission information, read-backed phasing, and joint analysis of each trio. Our final set of 100 full MHC haplotypes have <2% missing data and >92% of all variants phased. We recently reported that we found a total of 701 kb of novel sequence in these haplotypes and that some of these segments are large (3–6 kb) and common in our haplotypes (present in 22%–26% of parental haplotypes) (Maretty et al. 2017). Here, we describe our method of assembly and phasing in detail and perform an evolutionary analysis of the resulting haplotypes.

## Results

### Assembly of 100 full MHC haplotypes

Our assembly approach was designed to circumvent the challenges in mapping short reads to a reference sequence. Through several steps, we leverage transmission information and read-backed phasing to create candidate haplotypes to which we can map reads. Because the candidate haplotypes were created from the reads themselves, subsequent mapping is more successful than mapping to the reference genome, and phasing is improved. The procedure of mapping and phasing is iterated, as each inferred phased haplotype improves mapping and in turn phasing.

**Figure 1.**
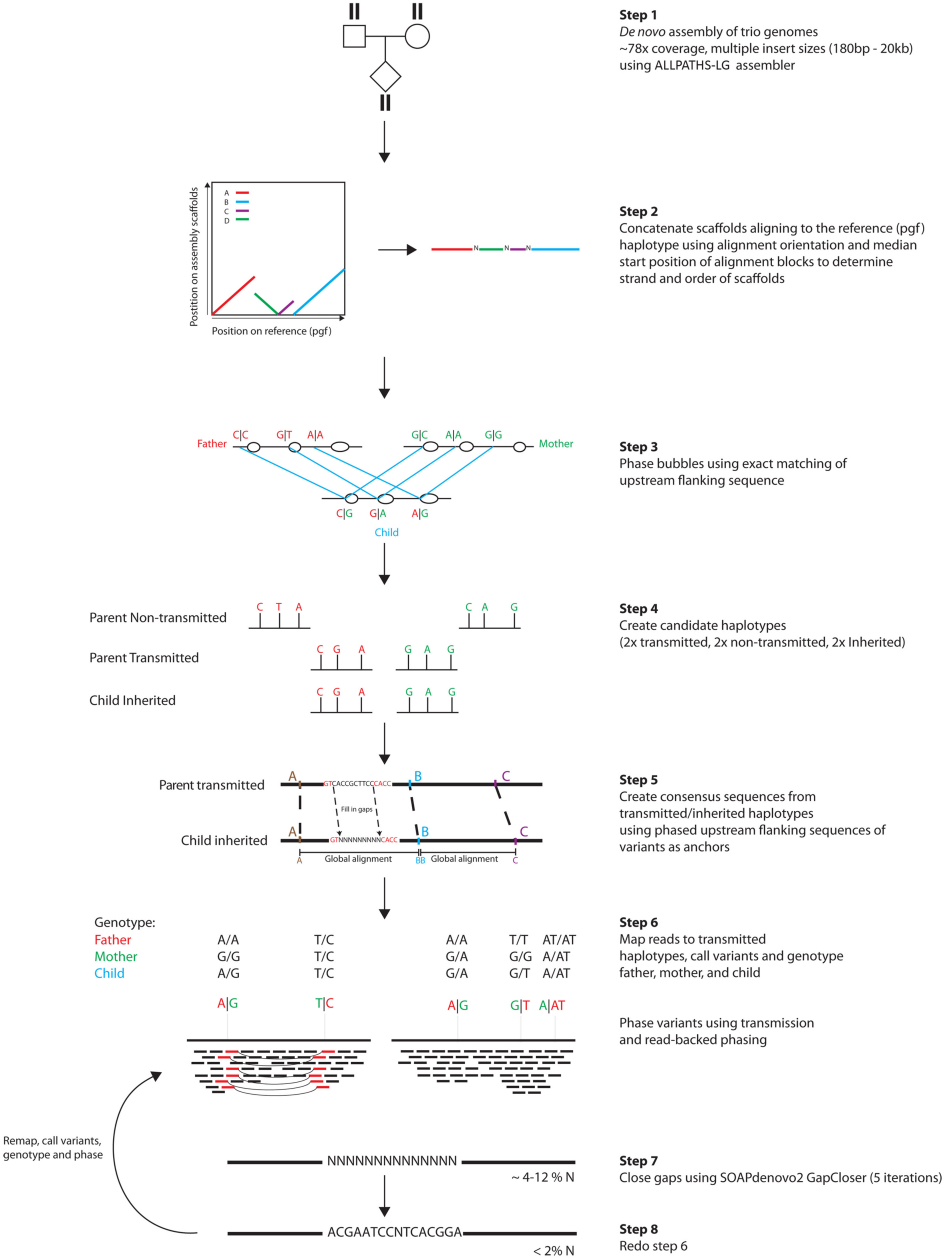
Assembly of 100 full MHC haplotypes. Schematic showing the construction of MHC haplotypes. Genomes in trios are de novo assembled using ALLPATHS-LG (step 1). Scaffolds larger than 50 kb mapping to the MHC are extracted and concatenated, creating diploid consensus scaffolds (step 2). Bubbles in the alignment graphs for individuals in the trio are mapped uniquely within the trio by exact matching of the sequence upstream of the bubbles (step 3). Global alignment between phased bubbles is used to create a consensus sequence between transmitted parental and inherited child haplotype sequences (steps 4 and 5). Reads from parents and child are then mapped to the consensus sequence, genotyped, and phased (step 6), gaps are closed (step 7), and reads are mapped again for another iteration of mapping, genotyping, and phasing (step 8).

Figure 1 shows a schematic of our pipeline. The starting point is a set of scaffolds for each individual, de novo assembled using ALLPATHS-LG (Gnerre et al. 2011) on genomes sequenced to 78× by multiple insert size libraries (Maretty et al. 2017).

We extracted scaffolds mapping with at least 50 kb to the MHC region (the number of scaffolds ranges from 1 to 8 across individuals) (Supplemental Fig. S1a) and concatenated these to create diploid consensus scaffolds including bubbles in the assembly graph (step 2). For each trio, >77% of the bubbles in the alignment graphs were phased without Mendelian violation using the sequence immediately upstream of and downstream from each bubble to find exact matches within the trio (step 3). After phasing, we created a sequence for each nontransmitted parental haplotype and created a consensus sequence between transmitted parental haplotypes and inherited child haplotypes by multiple global alignments of segments between phased bubbles (steps 4 and 5). We then mapped reads to the transmitted (consensus) haplotypes and genotyped and phased them using transmission information and read-backed phasing (step 6). We then closed gaps to obtain full-length haplotypes with <2% gaps (step 7) (Supplemental Fig. S1b). A second iteration of mapping, genotyping, and phasing resulted in phasing of >92% of the variants in the transmitted haplotypes, of which >80% were mapped back to the nontransmitted haplotype using exact matching (step 8) (Supplemental Fig. S1c). We evaluated the accuracy of variant calling and phasing by cloning and Sanger sequencing of five clones from 75 random fragments from highly polymorphic regions containing between two and 10 variants (204 variants in total). We found a validation rate of 86% (for details, see Supplemental Table S1) for the phase of the variants.

We used simulations to further evaluate the power and accuracy of our approach by simulating reads in an artificial trio with known MHC haplotypes, reconstructing the haplotypes using our pipeline, and comparing these to the original haplotypes. We simulated reads from a trio with four of the different reference haplotypes—pgf and mcf in the mother, cox and qbl in the father, and pgf and cox in the child. Reads were simulated to exactly reflect the coverage, insert size distribution, and error profile as our own sequencing. De novo assembly and inference of phased haplotypes were then done in exactly the same way as for the real data using our pipeline outlined in Figure 1; we then investigated whether we could separately recover the cox and the pgf haplotypes in the child. Supplemental Figure S2, a and b, shows that although the initial assembly in the child is a mixture of the two haplotypes, the final haplotypes generally align over the whole region with pgf and cox, respectively, showing that the pipeline has phased them. We found that 91.6% of the haplotypes aligned to the correct haplotypes (Supplemental Fig. S2b), and the lengths of incorrectly phased segments were generally very short compared to the correctly phased segments (Supplemental Fig. S2c). Because collapse of paralogous or repetitive sequence might be a likely error mode in the assembled haplotypes (Alkan et al. 2011), we calculated the content of *Alu* and LINE-1 repetitive elements as a measure of the amount of collapsed repetitive sequence in the eight reference haplotypes, our simulated haplotypes, and our 100 new haplotypes. We found that both our simulated and new haplotypes have *Alu* and LINE-1 content similar to the reference haplotype, and the variation among haplotypes in *Alu* and LINE-1 content is considerable (Supplemental Fig. S3). The six incomplete reference haplotypes all show a strong deficiency in these elements.

The length of the 100 individual haplotypes range from 4.5 to 5.2 Mb (Supplemental Fig. S1d), and missing data in the haplotypes range between 0.2% and 2% (Supplemental Fig. S1e). The distribution of missing data over the MHC region is shown in Supplemental Figure S4. It also shows that there are large blocks of missing data in six of the eight haplotypes supplied with the reference genome.

**Figure 2.**
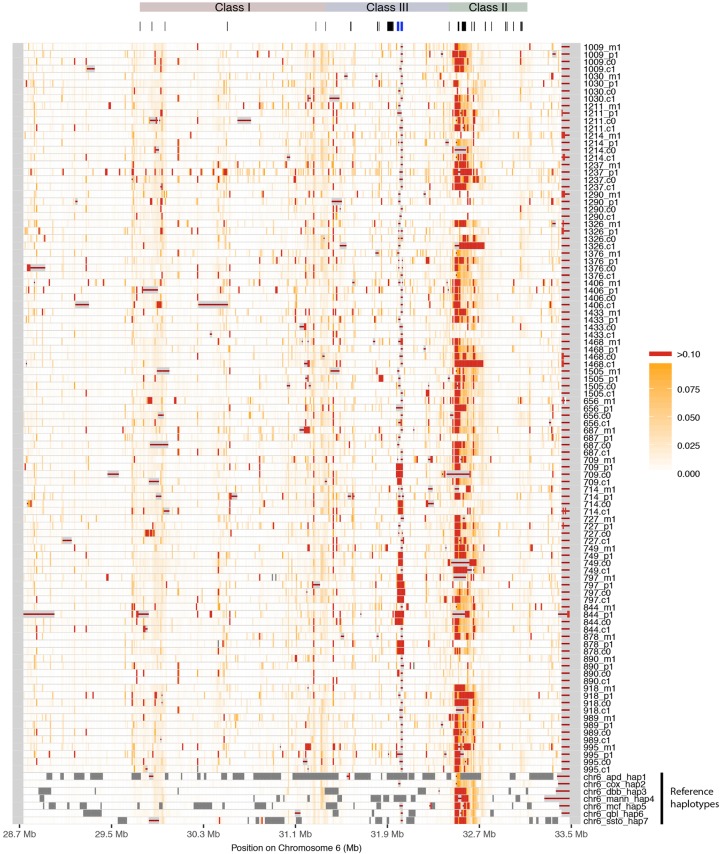
Differences between MHC haplotypes and reference pgf. The new haplotypes and the seven alternative reference haplotypes were aligned to the reference pgf haplotype through pairwise alignment, and the percentage of pairwise differences was calculated in bins of 10 kb, shown here in white (low) to red (high). Dark gray bins contain >50% missing data (i.e., Ns); bins with red line lack alignment blocks. The region classes and important genes such as the classical loci are shown *above*. *C4A* and *C4B* are marked in blue.

To visualize the differences among our haplotypes, we aligned them one by one to the pgf and cox reference haplotypes from hg38 using MAFFT (Katoh and Standley 2013) and scored the percentage of differences in the alignment in 10-kb windows along the MHC. Figure 2 shows a heat plot of differences with the pgf haplotype (a similar heat plot against cox is found in Supplemental Fig. S5).

The six existing haplotypes from the human reference genome are included for comparison, showing that these contain many sequencing gaps. In contrast, our new haplotypes contain fewer sequencing gaps (Supplemental Fig. S4). The diversity is variable but generally very high across the region. In the proximal part of the class II region, diversity is so high that alignment becomes unreliable, explaining well why mapping-based approaches fail in this region, which is also among the most important in association mapping studies. We noticed that alignment to the reference haplotype (pgf) near the *C4A* and *C4B* genes (Chr 6: 31982024–32002681, Chr 6: 32014762–32035418, blue gene markers in Fig. 2), known to harbor common structural variation and to be associated with several diseases, is poor for most haplotypes including the alternative reference haplotypes. When we align to the cox haplotype, we can improve alignment in this region significantly for most haplotypes (Supplemental Fig. S5); however, for some haplotypes, alignment is still poor. We conclude that identification of structural variation in this region by alignment to the reference haplotype is not reliable (Dilthey et al. 2015; Sekar et al. 2016), and new approaches such as graph-based methods are needed to fully exploit our new haplotypes for mapping and imputation in the most complex parts of the extended MHC region.

Because all of our new haplotypes come from the Danish population, which genetically is quite homogenous (Athanasiadis et al. 2016), we wanted to assess the extent to which our haplotypes represent global MHC diversity, because this is an important aspect to consider when looking for disease associations in the MHC. To investigate this, we sampled five random diploid MHC regions from each of the 26 populations in The 1000 Genomes Project (The 1000 Genomes Project Consortium 2015) and compared the sampled regions with our new haplotypes using principal component analysis and constructing a neighbor-joining tree based on the distance matrix computed from the data (Supplemental Fig. S6). We find that our haplotypes well represent global diversity in the MHC region, which fit with our prior expectation, since most MHC diversity is likely to be old and maintained by balancing selection over much longer time spans (millions of years) than the divergence of human populations (<100,000 yr).

### Population genetics of the MHC

For population genetics analyses, we chose to focus on the haplotypes with the most phased variants and the least amount of sequence gaps—the 50 haplotypes transmitted to the children. To obtain a reliable variant call set in reference genome coordinates, we aligned against hg38 and used the AsmVar pipeline (Liu et al. 2015) to produce a large and error-prone candidate set of variants called from the alignment. This candidate set was then evaluated in all 25 children using the BayesTyper application (JA Sibbesen, L Maretty, The Genome Denmark Consortium, A Krogh, in prep.), which assign genotype probabilities from comparing *k*-mer profiles from the reads with *k*-mers present in the reference and in the candidate set of variants. From a candidate set of 193,170 SNV and 32,002 structural variants, we call and genotype 50,170 SNVs and 5742 indels and complex variants. In contrast, we only found a total of 16,702 variants in our initial analysis in which we used the unphased scaffolds in the MHC region for variant calling.

As a test of the accuracy of this call set, we compared our inferred genotypes to the genotypes called by a SNP chip (HumanCoreExome BeadChip v.1.0) on the same individuals. In our samples, 2475 SNPs were polymorphic and genotyped in all individuals in our call set and on the chip. We found an overall concordance of 97.5%, which reflects a concordance >99% for most of the MHC region and a few less concordant regions due to very high levels of polymorphism likely to affect both our inference and the accuracy of the SNP chip (Supplemental Fig. S7).

Comparing to dbSNP, we find that most SNVs in our call set are known due to the large number of previous targeted investigations of the MHC (only 9.11% of SNV variants are novel) (Table 1). In strong contrast, most indels and complex variants >50 bp we identify are novel (25.00% for deletions and 98.37% for insertions, 99.56% for complex variants) (Table 1), suggesting they have been missed in previous studies.

**Table 1.**
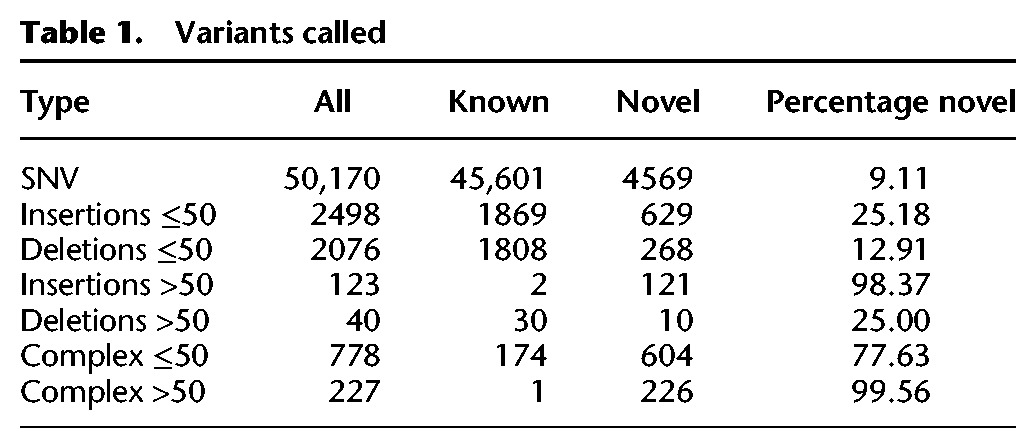
Variants called

Because of the complexity and inaccessibility of the MHC region, most previous studies have focused on specific regions of the MHC. Our new haplotypes allowed us to gain a more global view of the region.

We calculated the folded site frequency spectrum for nine classical HLA genes (*HLA-A*, *HLA-C*, *HLA-B*, *HLA-DRA*, *HLA-DRB1*, *HLA-DQA1*, *HLA-DQB1*, *HLA-DPA1*, *HLA-DPB1*), the entire MHC region, and for the entire genome (Supplemental Fig. S8). The site frequency spectrum is shifted toward more common variants in the whole region and in the classical HLA genes in particular when compared to the rest of the genome.

**Figure 3.**
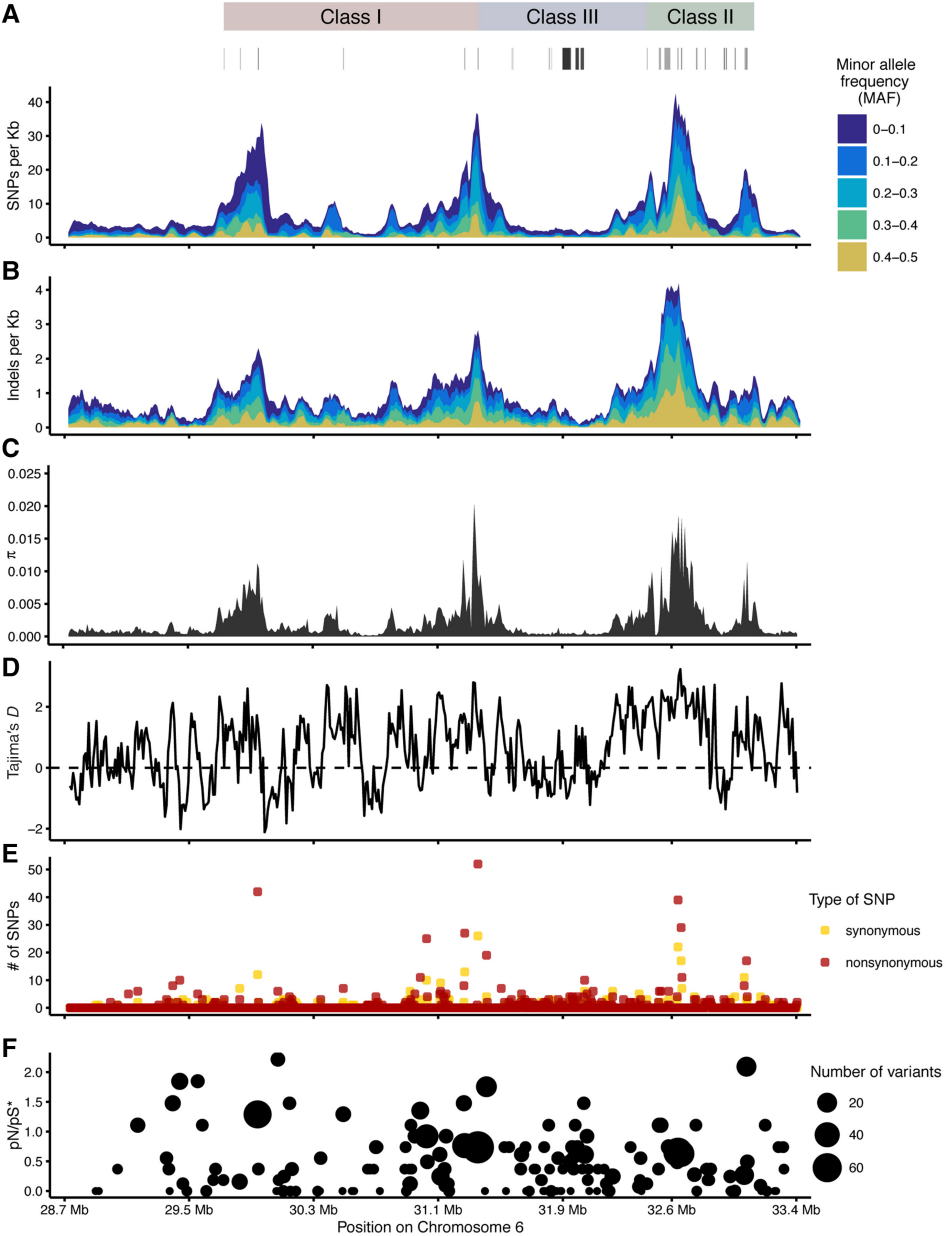
Variation and population genetics. (*A*,*B*) Number of SNVs and indels across the MHC region in 50-kb sliding window (step 10 kb). (*C*) Nucleotide diversity (π) and (*D*) Tajima's *D* were calculated in 5-kb sliding windows (step 1 kb). (*E*,*F*) Count of nonsynonymous and synonymous SNVs across the MHC region and pN/pS estimated assuming 73% and 27% of sites to be nonsynonymous and synonymous, respectively, calculated as the proportions in the reference pgf haplotype. The MHC classes and important genes, such as classical HLA genes, are marked *above*.

Figure 3 shows SNV and indel variation along the MHC region for the 50 haplotypes. Nucleotide diversity is far above genome average in three broad regions, where the folded site frequency spectrum of SNVs is also shifted to intermediate frequencies. Indels occur with higher relative frequency outside classical loci compared to SNVs and with higher minor allele frequencies also (Fig. 3B).

We observe Tajima's *D* statistics above genome-wide values extending from the classical loci along with an increase in the proportion of nonsynonymous variants, consistent with linkage to sites under balancing selection in classical MHC genes (Fig. 3D–F; Supplemental Fig. S9).

**Figure 4.**
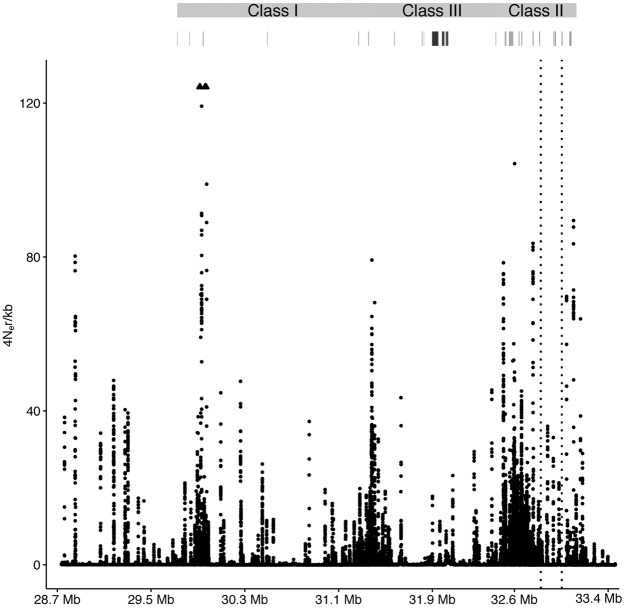
Recombination across the MHC region. Recombination rate estimated across the MHC region. Arrowheads point *up* toward two outliers that were removed for better visualization of the rest of the region.

The recombination rate inferred using *LDhat* (Auton and McVean 2007) is highly variable across the entire MHC region, with recombination rate hotspots interspersed with regions of very low recombination rate (Fig. 4A). We find no strong overall correlation between gene density and recombination rate, but in the most gene dense part of the class III region, we find long sequence stretches with low recombination rate. We find a high recombination rate in classical loci but also observe a high recombination rate outside classical loci, especially upstream of the Class I region. Jeffreys et al. (2001) determined recombination in a 200-kb region of the MHC using sperm typing, and our results show concordant peaks in recombination rate in this region, supporting the accuracy of our recombination map inference (Supplemental Fig. S10).

**Figure 5.**
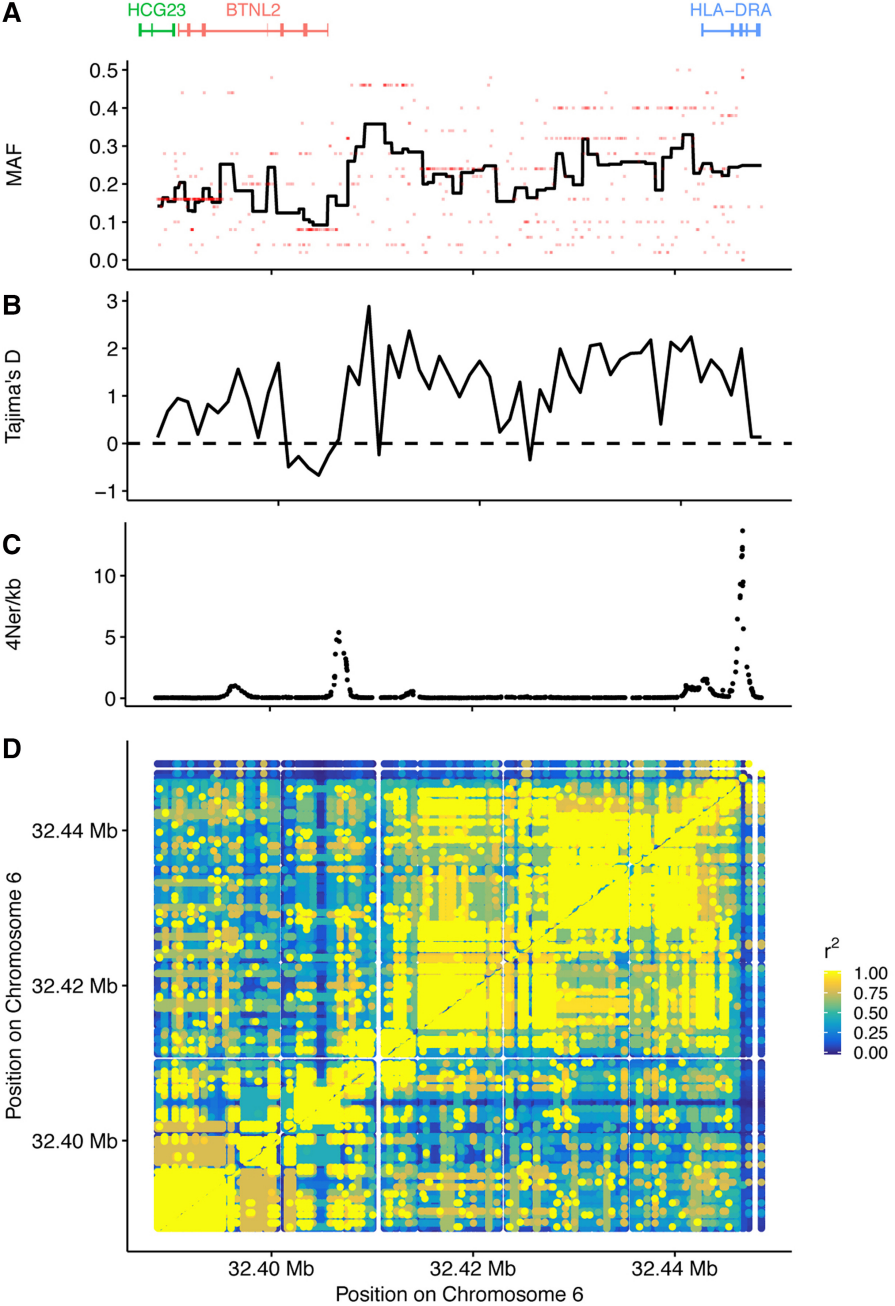
LD patterns and selection upstream of *HLA-DRA*. (*A*) Average minor allele frequencies (MAF) across the region. The red dots are the MAF of the variants, and the line shows the average MAF in bins of 10 variants. (*B*) Tajima's *D* statistic calculated in 1-kb bins. (*C*) Recombination rate estimate. (*D*) In a 60-kb region upstream of the *HLA-DRA* gene, the *r*^2^ statistics was calculated.

In order to study potential consequences on linked diversity of balancing selection acting in the MHC region, we first chose to focus on a region 60 kb upstream of and including the classical *HLA-DRA* gene (Fig. 5), which has been shown to be under balancing selection in the CEU populations (DeGiorgio et al. 2014), to see if we could detect balancing selection and to what extent these signatures extend away from the locus. We detected strong LD extending upstream of the gene (Fig. 5D), and although the average minor allele frequency of variants decays slowly moving away from this gene, we still see high minor allele frequencies in neighboring genes (Fig. 5A) along with a positive Tajima's *D* across almost the entire region (Fig. 5B). These observations are also reflected in the estimated recombination rate in the region (Fig. 5C). Although we see a minor peak in recombination rate between the genes, recombination rate is generally much lower compared to the entire region (Fig. 4). These observations suggest that balancing selection cause increased frequency of variation in genes linked to the classical *HLA-DRA* gene.

**Figure 6.**
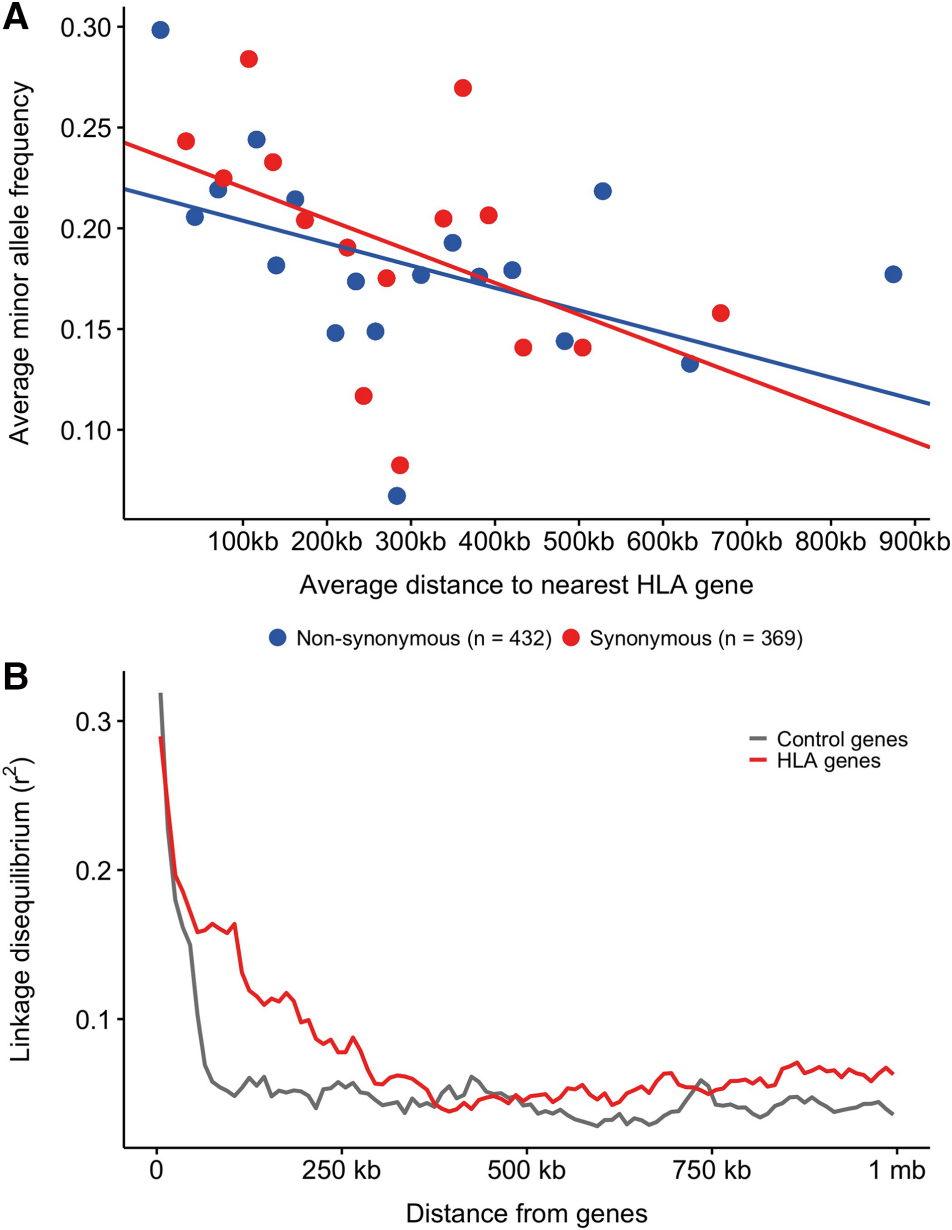
Linked selection. (*A*) Average minor allele frequencies of nonsynonymous (blue, *n* = 432, *P*-value <0.01) and synonymous variants (red, *n* = 369, *P*-value <0.001) were calculated in bins of 25 variant sites and plotted as a function of the average distance of those 25 variants to the nearest classical HLA gene (*HLA-A*, *HLA-C*, *HLA-B*, *HLA-DRA*, *HLA-DRB1*, *HLA-DQA1*, *HLA-DQB1*, *HLA-DPA1*, *HLA-DPB1*). Variants within the classical MHC genes are not included. A linear regression was fitted for each variant type on the nonbinned data. (*B*) Linkage disequilibrium (*r*^2^) calculated for all pairs of SNPs in either classical HLA genes (red) or control genes (gray) and all other SNPs in the MHC region are shown here as a function of distance from the genes.

We then decided to test whether this effect could be detected in other HLA genes known to be under balancing selection. In order to study the importance of selection and the frequency of coding variants in linked genes in general, we calculated the average minor allele frequency (MAF) of synonymous and nonsynonymous variants as a function of distance to the closest of nine HLA genes (classical HLA loci) previously shown to be under balancing selection (DeGiorgio et al. 2014). Figure 6A shows a gradual decline in minor allele frequency for both synonymous and nonsynonymous variants away from classical genes, which stretches over >100 kb.

These results are in line with the findings of Lenz et al. (2016) based on a much larger sample of exome-captured genes in the MHC region that balancing selection at the HLA genes shelters nonsynonymous variation of potential detrimental effects and/or of relevance for association findings in nearby genes. As a control, we randomly selected nine genes from the MHC region and compared the same metric but found no significant correlation between MAF and distance to the nearest control gene for synonymous variants and, although significant for nonsynonymous variants, the slope was in the opposite direction, i.e., positive (Supplemental Fig. S11). If balancing selection causes the increase in MAF, we would expect to see increased linkage disequilibrium (LD) near these HLA genes. We therefore calculated LD as a function of distance to the same HLA genes and found that LD indeed is high near HLA genes and extend up to hundreds of kilobases from the genes (Fig. 6B, red line). As a control, we selected nine genes in the genome, chosen randomly, but matched in length with a classical HLA gene, so a control gene of similar length matched each classical HLA gene. In contrast to the HLA genes we saw a much more rapid decay in LD moving away from control genes (Fig. 6B, gray line) in line with the overall decay of LD in the human genome.

These observations suggest that linked selection keeps variants in other genes at higher frequency with potential detrimental effects if some of these variants have a direct effect on fitness.

## Discussion

Our ability to assemble highly accurate full MHC haplotypes has allowed us to present a global view of the variation along this important region of the human genome. The preponderance of new structural variation shows that de novo assembly is necessary in order to catalog the full variation in the region. The 100 haplotypes we release should have immediate use as an imputation panel for deciphering the causative variants of genome-wide association studies (GWAS) reported in a large number of studies.

A recent advance in genome inference in the MHC region is the construction of population reference graphs (Dilthey et al. 2015). Population reference graphs tie together variant sequence such as MHC alleles from the IMGT/HLA database and variants from The 1000 Genomes Project (The 1000 Genomes Project Consortium 2015) with full-length haplotypes. The IMGT/HLA database has accumulated 17,166 MHC alleles (release 3.29.0, July 10, 2017) since the first release (1.0) in 1998. Excluding the highly diverse *HLA-DRB* genes (*HLA-DRB1*, *HLA-DRB5*), in which we find 420 novel variants, we only find 317 new variants in the classical MHC genes. However, the haplotype sequences that constitute the majority of the graph outside classical loci are currently constructed from only eight reference haplotypes. Considering the amount of missing data in six of the eight reference haplotypes, we anticipate that population reference graphs of the MHC region using our 100 novel haplotypes will improve inference and variant discovery, particularly outside the classical loci such as the *C4A*/*C4B* genes, in which imputation is otherwise limited due to poor alignment. Current implementations of MHC population reference graphs rely on multiple sequence alignment in blocks of sequence between and within classical loci across the MHC region. This approach works especially well for studying small variants but is limited in the ability to detect new structural variation, because the quality of the graph is largely reliant on the quality of the sequence alignment, which can be poor in some regions such as near the *C4* genes.

In order to capture structural variation at classical loci, graph methods should not rely on alignment to a reference sequence based on annotation. Better would be to construct a graph that relies on detection of nonvarying sequences in the MHC region that are shared among haplotypes instead of using annotation of classical loci. This allows for a graph structure in which all structural variation is retained in the graph and can be described by paths through common anchor sequences. Including a reference sequence in the graph construction will allow annotation of the variants without biasing variant identification toward the reference.

A recent study used a capture array and deep sequencing of the complete MHC region in 20,635 individuals of Han Chinese ancestry (Zhou et al. 2016). Among the 224,872 reported SNVs, only 29,429 are common (MAF>5%) and only 0.19% of the common SNVs are novel. In contrast, we report 44,370 common SNVs of which 6.06% are novel, suggesting that the full assembly allows us to access variation not easily captured by an array.

The approaches to studying variation in the MHC have different advantages and drawbacks. For instance, although capture arrays can accurately detect much of the variation in the MHC, they are by construction limited in the amount of new variation that they can find. Similarly, the population reference graphs can greatly improve inference using mapping-based approaches but are constructed from known variation and depend largely on accurate alignment to known annotations. Recently, a novel method for capturing and sequencing the MHC based on homozygous cell lines was used to accurately determine the sequence of 95 MHC haplotypes, including the highly polymorphic class I and class II genes and the structurally variant *C4* genes (Norman et al. 2017). These haplotypes are likely to be better resolved than our haplotypes in some of the most polymorphic regions, but it is important to notice that they are built only from sequence captured by probes in the region, potentially missing novel sequence. It was also noted by Norman et al. (2017) that the MHC haplotypes were not selected randomly and are therefore unsuitable for formal analysis of linkage disequilibrium. Our haplotypes are remarkable because they are built from de novo assemblies and phased essentially without the reference genome. Our survey of variation in the children based on alignment against the reference is suboptimal for complex variation since this is not all included in the LAST (Kiełbasa et al. 2011) ⇒ AsmVar (Liu et al. 2015) ⇒ BayesTyper (JA Sibbesen, L Maretty, The Genome Denmark Consortium, A Krogh, in prep.) pipeline that we have used. When graph-based methods mature (Paten et al. 2017), our data will also allow the large novel indels and complex variants to be incorporated and imputed into genotype and short read studies of the MHC region. This includes the novel common insertions of >700 kb novel sequence in fragments sometimes exceeding 5 kb that we reported from *k*-mer profiling in Maretty et al. (2017).

Importantly, despite this caveat in our method, our haplotypes can be utilized to improve the shortcomings of other methods. For instance, the addition of our haplotypes to population reference graphs will make it possible to study novel sequences using mapping-based approaches and will enable us to place them more accurately in the reference genome. It will also enable design of new capture arrays and probes to access more of the MHC region and perhaps gain more insight into how much of this previously unknown sequence is common, how much is polymorphic, and whether any of these are functional.

Our evolutionary analyses indicate that the abundant balancing selection affects a large part of the region, keeping variation linked to classical variation at a very high frequency. We speculate that some of this linked variation may be deleterious but sheltered by strong balancing selection and therefore also contain some of the disease associations reported.

## Methods

### Data

The parent–offspring trios (mother–father–child) in the Danish Pan-Genome were selected from the Copenhagen Family Bank (Eiberg et al. 1989; Maretty et al. 2017). The study protocol was reviewed and approved by The Danish National Committee on Health Research Ethics (file number 1210920, submission numbers 36615 and 38259).

### Phasing of MHC haplotypes

We constructed haplotypes of the whole MHC region using ALLPATHS-LG (Gnerre et al. 2011) scaffolds as the starting point, including variants in FASTG format (http://fastg.sourceforge.net/FASTG_Spec_v1.00.pdf) from scaffolds.

We aligned scaffolds to the reference genome (hg38) using the LAST (Kiełbasa et al. 2011) aligner with the following parameters: lastal –e25 –v –q3 –j4 –m 100 | last-split –s35 –v –m 0.01. The parameters were optimized for high sensitivity alignment with a relaxed error threshold, allowing alignment of more dissimilar scaffolds. We then extracted scaffolds of at least 50 kb mapping to the MHC region from the assembly graphs. The entire scaffolds were used and not only the parts mapping to the reference.

In order to determine the orientation and order of the scaffolds aligning to the MHC region, we calculated the median of the start position of each scaffold alignment to the reference sequence. Alignment blocks of less than half the size of the greatest alignment block were excluded. We determined the order of the scaffolds from the median start position and determined the orientation by the sum of the lengths of scaffolds aligning in either sense or antisense orientation. Scaffolds aligning in antisense orientation were reverse complemented. The start and end of the region was defined as 1 Mb upstream of the major histocompatibility complex, class I, F (*HLA-F*), and 1kb downstream from the kinesin-like protein (*KIFC1*), roughly corresponding to the range defined in the reference haplotype (pgf). The sequences of *HLA-F* and *KIFC1* were used to perform BLAST (Altschul et al. 1990) (BLASTN) against the first and last scaffold in the order, respectively, and the starting position of each gene determined from the highest scoring hit. The scaffolds were then trimmed accordingly and finally concatenated to create full-length MHC scaffolds. A gap of length one (“N”) was added in between the scaffolds to indicate the break between scaffolds.

We determined positions of variant sites from the graph within the trio by exact matching of 40 bp upstream of each variant. Upstream flanking sequences (UFS) of length 40 bases were extracted for each variant extracted from the assembly graphs. For each individual in a parent–offspring trio, the UFS was used to perform exact matching against each individual in the trio. More specifically, only uniquely matching positions were kept from each individual, discarding multiple mappings of an UFS to different positions in an individual or unique matching of UFS from different individuals to the same position in an individual. Likewise, the reverse complements to the upstream flanking sequence (RUFS) were used to perform exact matching in order to capture putative inversion events.

For variable sites, we genotyped each individual in a trio by exact matching of UFS and RUFS. Sites with missing data for one or more individuals were excluded. For each position, variants found in the parents were added to the offspring variant call set. Each individual in the trio was then genotyped either by the variants from their own call set or by lookup in the sequence at the given position. Biallelic variants were phased using transmission information within the trio. Subsequently, sequences were created for each of the six haplotypes, i.e., the transmitted and nontransmitted haplotypes from father and mother, respectively, and the child haplotypes inherited from father and mother, respectively. The variant call sets were then updated to account for changes in variant lengths.

We constructed consensus sequences for each parent–offspring haplotype using global alignment between all pairwise sets of phased variants. Haplotypes were refined by first mapping reads to the four haplotypes within each trio using BWA-MEM version 0.7.5a (Li and Durbin 2009), then calling variants with Platypus, version 0.7.9.1 (Rimmer et al. 2014), and finally phasing variants that passed quality control by determining the parent of origin (PoO) of alternative alleles (for details, see Maretty et al. 2017). Gaps in the haplotypes were closed using the GapCloser module from SOAPdenovo2 (Luo et al. 2012) through five iterations of adding one read library at a time. After gap closing, all transmitted haplotypes were submitted to remapping, variant calling, and phasing as described above. Variant positions in nontransmitted haplotypes were mapped by pairwise alignment to the transmitted haplotypes.

### Variant calling and variant annotation

All transmitted haplotypes were aligned to hg38 using the LAST (Kiełbasa et al. 2011) aligner. The AsmVar pipeline (Liu et al. 2015) was used to create a candidate set of genotypes from the two haplotypes from each individual. BayesTyper (JA Sibbesen, L Maretty, The Genome Denmark Consortium, A Krogh, in prep.) was used to call variants from the candidate set of variants; phasing was restored by using the allele call origin INFO field from AsmVar (Liu et al. 2015) and removing any variants discordant in respect to phasing and allele call origin. Alleles with allele call probabilities greater than 0 were kept to create a more refined call set. Genotyping and phase restoration was then performed again for all individuals in a joint call set in order to rescue missed genotypes.

Variants were annotated using ANNOVAR (Yang and Wang 2015) and variants from dbSNP (release 142) and The 1000 Genomes Project (phase 3) (The 1000 Genomes Project Consortium 2015). Variants were classified as either known or novel. Variants were considered novel if not annotated in dbSNP (release 142) or The 1000 Genomes Project (phase 3) (The 1000 Genomes Project Consortium 2015).

### Pairwise alignment to reference haplotypes

Alignment of novel and alternative reference haplotypes to the pgf and cox reference haplotypes was performed using MAFFT (7.245) (Katoh and Standley 2013) with the parameters --fft and --memsave. Alignments that failed in >20% of the length of the reference were removed. N-content and number of pairwise differences were counted in bins of 10 kb across the entire region.

### Population genetics

Nucleotide diversity, Tajima's *D*, *r*^2^, and minor allele frequencies were computed using VCFtools version 0.1.14 (Danecek et al. 2011). Nonsynonymous and synonymous variants were counted in coding regions, and pN and pS were estimated using the fraction of nonsynonymous (0.73) and synonymous (0.27) sites calculated from the reference (pgf).

### PCA plot and NJ tree of HLA haplotypes

We merged our vcf file (25 individuals) with all individuals from The 1000 Genomes Project (The 1000 Genomes Project Consortium 2015) using VCFtools version 0.1.14 (Danecek et al. 2011). Subsequent analysis was done in R (version 3.4.0) (R Core Team 2014) using the packages SNPRelate (Zheng et al. 2012) and APE (Paradis et al. 2004). All 25 Danish individuals and five random individuals from each of The 1000 Genomes Project (The 1000 Genomes Project Consortium 2015) populations was selected, and SNPs with >5% missing data were removed. Standard PCA plot was made using the function snpgdsPCA() and the Neighbour-Joining tree was built from the distance matrix created by the function snpgdsDiss().

### Genotype concordance

The HumanCoreExome BeadChip v.1.0 was used to genotype the individuals using the HiScan system (Illumina). Genotypes were called using GenomeStudio software (v2011.1; Illumina). Concordance was calculated from all sites (*n* = 2475) genotyped by the chip and BayesTyper (JA Sibbesen, L Maretty, The Genome Denmark Consortium, A Krogh, in prep.) in all individuals.

### Linked selection and LD decay

We calculated the minor allele frequency of all synonymous and nonsynonymous variants from our call set in all genes in the MHC region. For each of these variants, we calculated the distance to the nearest classical HLA gene (*HLA-A*, *HLA-C*, *HLA-B*, *HLA-DRA*, *HLA-DRB1*, *HLA-DQA1*, *HLA-DQB1*, *HLA-DPA1*, *HLA-DPB1*). We then made a linear regression on the minor allele frequency and distance to the nearest classical locus. We then binned all variants in bins of 25 variants, except those within the classical HLA genes. For each bin, we calculated the average minor allele frequency and the average distance to the nearest classical HLA gene and plotted this for better visualization. We did the same for nine randomly selected control genes (*HCG14*, *VWA7*, *LY6G6C*, *CSNK2B*, *DAXX*, *MIR6832*, *NELFE*, *SAPCD1*, *TRIM39-RPP21*) from the MHC region to serve as a control.

LD was calculated for all pairs of SNPs in classical HLA genes (snp1) and all other SNPs (snp2) using VCFtools (Danecek et al. 2011) and the options --geno-r2-positions and –maf 0.05. All pairwise LD measures were summarized in 10-kb bins using the arithmetic mean. This procedure was then applied to a set of control genes (*GSTM3*, *D2HGDH*, *PDE6B*, *TUBGCP2*, *LRRC32*, *IRX5*, *RAB40B*, *SAFB2*, *SLC5A4*) that were randomly selected from all genes in the genome, but matched in length with the classical HLA loci, so each classical HLA gene was matched by a control gene of similar length.

### Recombination rate

The recombination rate was calculated using *rhomap* (*LDhat*) (Auton and McVean 2007) on the joint genotype call set using a likelihood table with *n* = 50 and *t* = 0.001 with a total of 1,010,000 iterations and a burn-in of 10,000 iterations. Samples of the chain were taken every 2500 iterations after the burn-in.

### Validation by simulation

We used ART (Huang et al. 2012) to generate read error models and quality profiles for each library size from the sequencing libraries of a randomly selected trio. We then designed a trio in which the father carries the cox and the qbl MHC haplotypes, the mother carries the pgf and the mcf MHC haplotypes, and the child carries the pgf and the cox haplotypes, such that we expect the father to have transmitted the cox haplotype and the mother to have transmitted the pgf haplotype. For each individual in this trio, we used ART (Huang et al. 2012) to simulate reads from the corresponding haplotypes with coverage matching the average coverage in the Danish trio data for each insert size, so that the simulated read coverage is similar to the real data. The reads were then used to de novo assemble the MHC region in each individual with ALLPATHS-LG (Gnerre et al. 2011) using the same settings and parameters used for the real data. The phasing pipeline was applied in order to phase the haplotypes; the results before and after phasing were evaluated by aligning the assembled sequences of the child to the reference haplotypes cox and pgf using LAST (Kiełbasa et al. 2011) to generate alignments and dot plots of the aligned segments.

### Repetitive element content

The content of repetitive elements was calculated using RepeatMasker (Smit et al. 1996–2010) to summarize the content of *Alu* and LINE-1 repetitive elements in the eight reference haplotypes (pgf, cox, mcf, qbl, mann, ssto, dbb, apd), the simulated haplotypes, and the 100 new haplotypes.

### Experimental validation

In order to validate the phase of our predicted variants, we performed clonal Sanger sequencing in five replicates per sample to capture a total of 75 regions containing between two and 10 variants (204 variants in total). We calculate the validation rate as the fraction of variants that have the same phase as we predicted out of the total number of variants of which we could correctly identify an allele.

## Data access

WGS data, Sanger sequencing data, and genotype data from this study have been submitted to the European Genome-phenome Archive (EGA; https://www.ebi.ac.uk/ega/home), which is hosted by the EBI, under accession number EGAS00001002108. Python scripts for the essential parts of the pipeline are available in the online Supplemental Materials and at https://github.com/jacobmjensen/phasemhc.

## Members of The Danish Pan-Genome Consortium

Lasse Maretty,^6^ Jacob Malte Jensen,^7^,^8^ Bent Petersen,^9^ Jonas Andreas Sibbesen,^6^ Siyang Liu,^6^,^10^ Palle Villesen,^7^,^8^,^11^ Laurits Skov,^7^,^8^ Kirstine Belling,^9^ Christian Theil Have,^12^ Jose M.G. Izarzugaza,^9^ Marie Grosjean,^9^ Jette Bork-Jensen,^12^ Jakob Grove,^8^,^13^,^14^ Thomas D. Als,^8^,^13^,^14^ Shujia Huang,^15^,^16^ Yuqi Chang,^15^ Ruiqi Xu,^10^ Weijian Ye,^10^ Junhua Rao,^10^ Xiaosen Guo,^15^,^17^ Jihua Sun,^10^,^12^ Hongzhi Cao,^15^ Chen Ye,^15^ Johan v Beusekom,^9^ Thomas Espeseth,^18^,^19^ Esben Flindt,^17^ Rune M. Friborg,^7^,^8^ Anders E. Halager,^7^,^8^ Stephanie Le Hellard,^19^,^20^ Christina M. Hultman,^21^ Francesco Lescai,^8^,^13^,^14^ Shengting Li,^8^,^13^,^14^ Ole Lund,^9^ Peter Løngren,^9^ Thomas Mailund,^7^,^8^ Maria Luisa Matey-Hernandez,^9^ Ole Mors,^8^,^11^,^14^ Christian N.S. Pedersen,^7^,^8^ Thomas Sicheritz-Pontén,^9^ Patrick Sullivan,^21^,^22^ Ali Syed,^9^ David Westergaard,^9^ Rachita Yadav,^9^ Ning Li,^10^ Xun Xu,^15^ Torben Hansen,^12^ Anders Krogh,^6^ Lars Bolund,^13^,^15^ Thorkild I.A. Sørensen,^12^,^23^,^24^ Oluf Pedersen,^12^ Ramneek Gupta,^9^ Simon Rasmussen,^9^ Søren Besenbacher,^7^,^11^ Anders D. Børglum,^8^,^13^,^14^ Jun Wang,^8^,^15^,^17^ Hans Eiberg,^25^ Karsten Kristiansen,^15^,^17^ Søren Brunak,^9^,^26^ Mikkel Heide Schierup^7^,^8^,^27^

## Supplementary Material

Supplemental Material

## Footnotes

6 Bioinformatics Centre, Department of Biology, University of Copenhagen, 2200 Copenhagen N, Denmark

7 Bioinformatics Research Centre, Aarhus University, 8000 Aarhus C, Denmark

8 iSEQ, Centre for Integrative Sequencing, Aarhus University, 8000 Aarhus C, Denmark

9 DTU Bioinformatics, Department of Bio and Health Informatics, Technical University of Denmark, Kemitorvet, 2800 Kongens Lyngby, Denmark

10 BGI-Europe, Ole Maaløes Vej 3, 2200 Copenhagen N, Denmark

11 Department of Clinical Medicine, Aarhus University, 8000 Aarhus C, Denmark

12 Novo Nordisk Foundation Center for Basic Metabolic Research, Section of Metabolic Genetics, University of Copenhagen, 2100 Copenhagen Ø, Denmark

13 Department of Biomedicine, Aarhus University, 8000 Aarhus C, Denmark

14 The Lundbeck Foundation Initiative for Integrative Psychiatric Research, iPSYCH, 8000 Aarhus, Denmark

15 BGI-Shenzhen, Shenzhen 518083, China

16 School of Bioscience and Biotechnology, South China University of Technology, Guangzhou 510006, China

17 Laboratory of Genomics and Molecular Biomedicine, Department of Biology, University of Copenhagen, 2100 Copenhagen Ø, Denmark

18 Department of Psychology, University of Oslo, 0317 Oslo, Norway

19 NORMENT, KG Jebsen Centre for Psychosis Research, Department of Clinical Science, University of Bergen, Bergen, 5021, Norway

20 Dr. E. Martens Research Group of Biological Psychiatry, Center for Medical Genetics and Molecular Medicine, Haukeland University Hospital, Bergen, 5021, Norway

21 Department of Medical Epidemiology and Biostatistics, Karolinska Institutet, Stockholm, 17177, Sweden

22 Department of Genetics, University of North Carolina, Chapel Hill, NC 27599-7264, USA

23 Department of Clinical Epidemiology (formerly Institute of Preventive Medicine), Bispebjerg and Frederiksberg Hospital, The Capital Region, Copenhagen, 2000 Frederiksberg, Denmark

24 Department of Public Health, Faculty of Health and Medical Sciences, University of Copenhagen, 2200 Copenhagen, Denmark

25 Department of Cellular and Molecular Medicine, University of Copenhagen, 2200 Copenhagen N, Denmark

26 Novo Nordisk Foundation Center for Protein Research, Faculty of Health and Medical Sciences, University of Copenhagen, 2200 Copenhagen N, Denmark

27 Department of Bioscience, Aarhus University, 8000 Aarhus C, Denmark
