# Supplementary material for: Assembly and analysis of 100 full MHC haplotypes from the Danish population
Source: Genome Res. 2017 Sep;27(9):1597–607. doi: 10.1101/gr.218891.116 (PMC5580718; doi:10.1101/gr.218891.116)
Supplement: Supplemental Material [file supp_27_9_1597__index.html]

Assembly and analysis of 100 full MHC haplotypes from the Danish population — Supplemental Material 

# Assembly and analysis of 100 full MHC haplotypes from the Danish population

## Supplemental Material

- Supplemental\_Materials.docx
- Supplemental\_Scripts.zip
