# Supplementary material for: Assembly and analysis of 100 full MHC haplotypes from the Danish population
Source: Genome Res. 2017 Sep;27(9):1597–607. doi: 10.1101/gr.218891.116 (PMC5580718; doi:10.1101/gr.218891.116)
Supplement: Supplemental Material [file supp_gr.218891.116_Supplemental_Materials.docx]

# Supplemental Material

*Assembly and analysis of 100 full MHC haplotypes from the Danish population*

Content

Supplemental Figures 2

Supplemental Figure S1. Scaffold statistics. 2

Supplemental Figure S2. Validation by simulation. 3

Supplemental Figure S3. *Alu*/LINE-1 content. 4

Supplemental Figure S4. Distribution of missing data. 5

Supplemental Figure S5. Differences between MHC haplotypes and reference cox. 6

Supplemental Figure S6. Global MHC diversity. 7

Supplemental Figure S7. Genotype concordance. 7

Supplemental Figure S8. Folded site frequency spectrum. 8

Supplemental Figure S9. Genome wide Tajima’s *D*. 9

Supplemental Figure S10. Recombination rate in 200kb region. 10

Supplemental Figure S11. Linked selection control. 11

Supplemental Tables 12

Supplemental Table S1. Validation. 12

The Danish Pan Genome Consortium 17

# Supplemental Figures

##
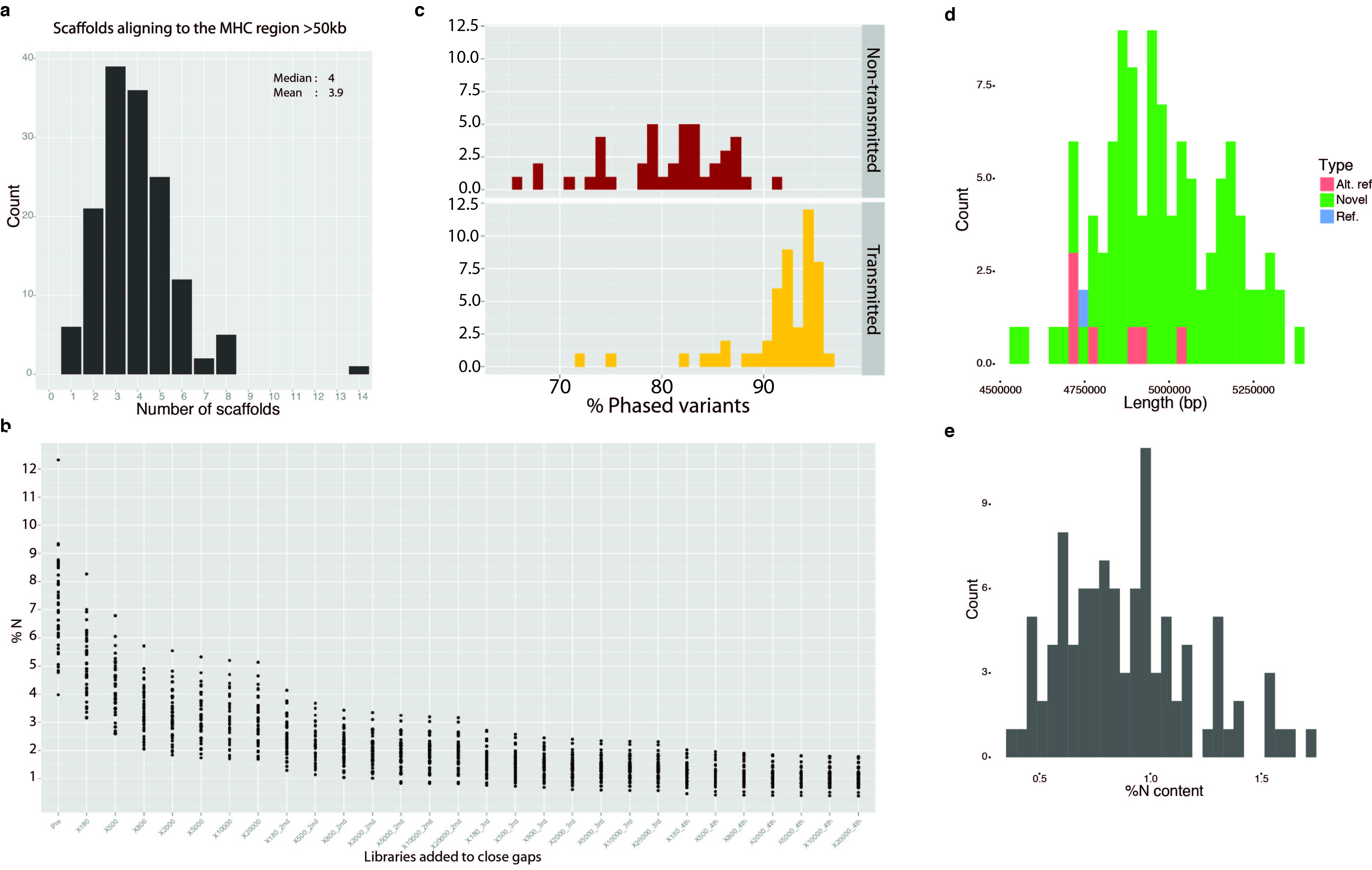
Supplemental Figure S1. Scaffold statistics.

(**A**) Number of scaffolds aligning to the MHC (>50kb). (**B**) Percentage of missing data (N’s) in haplotypes measured for each library added using the SOAPdenovo2 GapCloser module. (**C**) Percentage of phased variants in transmitted haplotypes (yellow, lower panel) and non-transmitted haplotypes (red, upper panel). (**D**) Distribution of novel (green), reference (blue) and alternative reference (red) haplotype lengths. (**E**) Distribution of missing data in novel haplotypes (N-content, i.e. percentage of haplotypes that are N’s).

## Supplemental Figure S2. Validation by simulation.

(a) Dotplot of child consensus sequence before phasing into haplotypes aligned to the cox and pgf reference haplotypes. (b) Dotplot of phased child haplotypes aligned to the cox and pgf reference haplotypes. Child_cox are the haplotypes in the child inherited from the father and mother, respectively, and therefore expected to be the cox and pgf haplotypes. (c) Length distribution of segments aligning to correct haplotype (blue) or wrong haplotype (red).


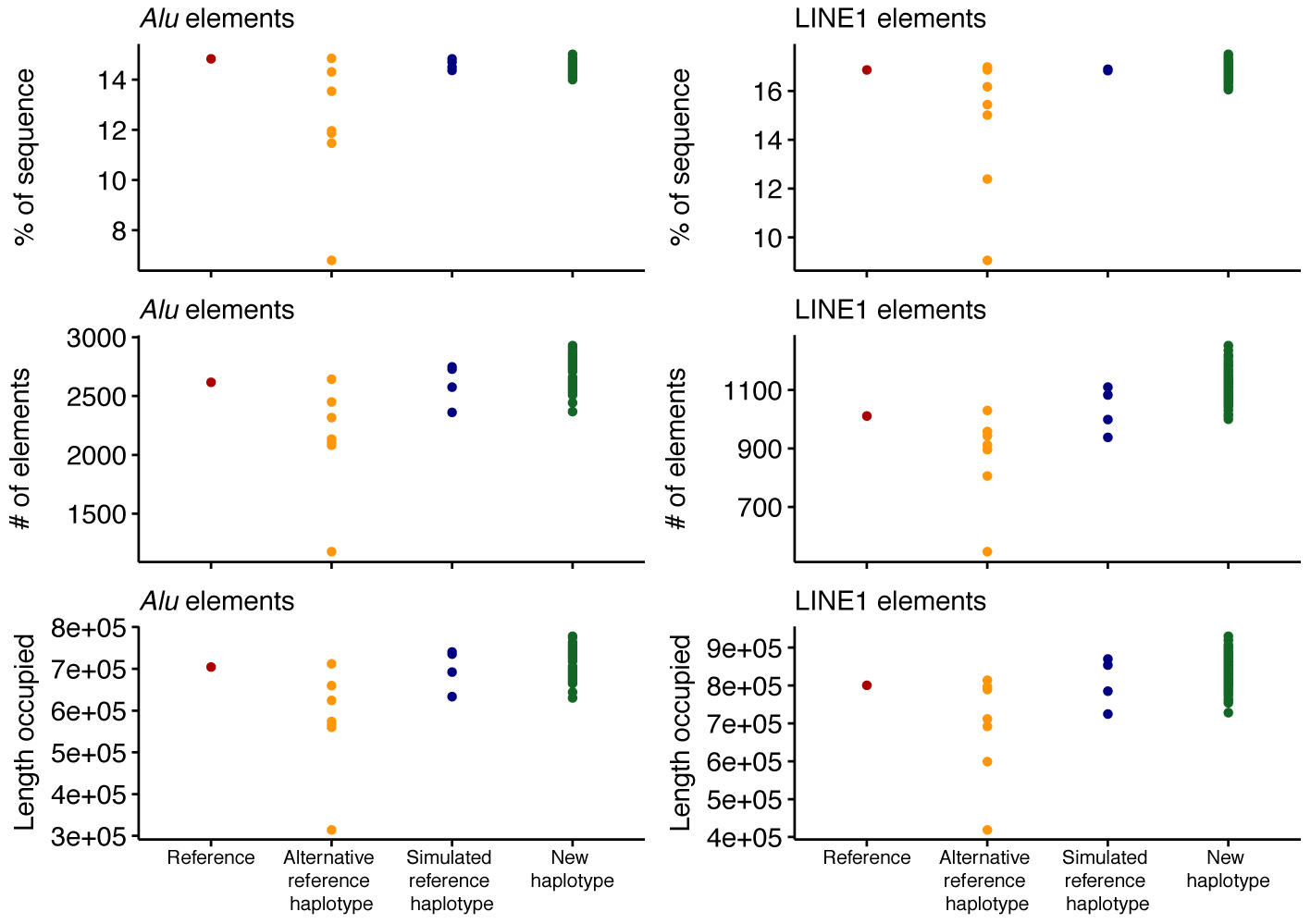


## Supplemental Figure S3. *Alu*/LINE-1 content.

*Alu*/LINE-1 contents in reference pgf haplotype (red), alternative reference haplotypes (yellow), simulated haplotypes (blue) and new haplotypes (green). (Top panels) Percentage of haplotypes that consists of *Alu* (left) and LINE1 (right) elements. (Middle panels) Number of *Alu* (left) and LINE1 (right) elements in haplotypes. (Bottom panels) Total length of *Alu* (left) and LINE1 elements in haplotypes.


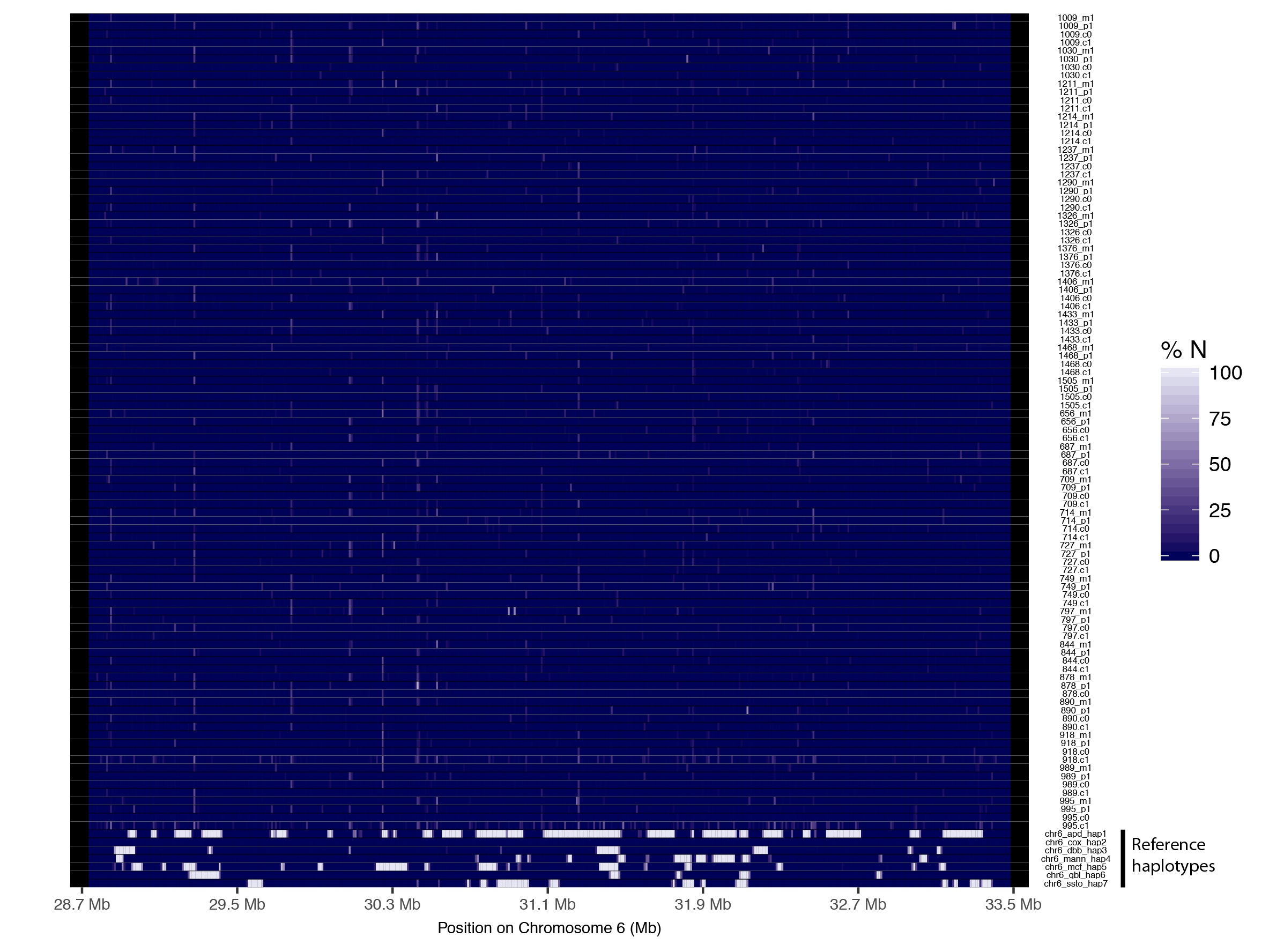


## Supplemental Figure S4. Distribution of missing data.

The heatmap shows the distribution of missing data (i.e. N’s) across the MHC region in novel haplotypes and the seven alternative reference haplotypes (apd, cox, dbb, mann, mcf, qbl and ssto; bottom seven rows). The haplotypes were aligned to the reference haplotype pgf through pairwise alignment and the percentage of missing data was calculated in bins of 10kb.

**
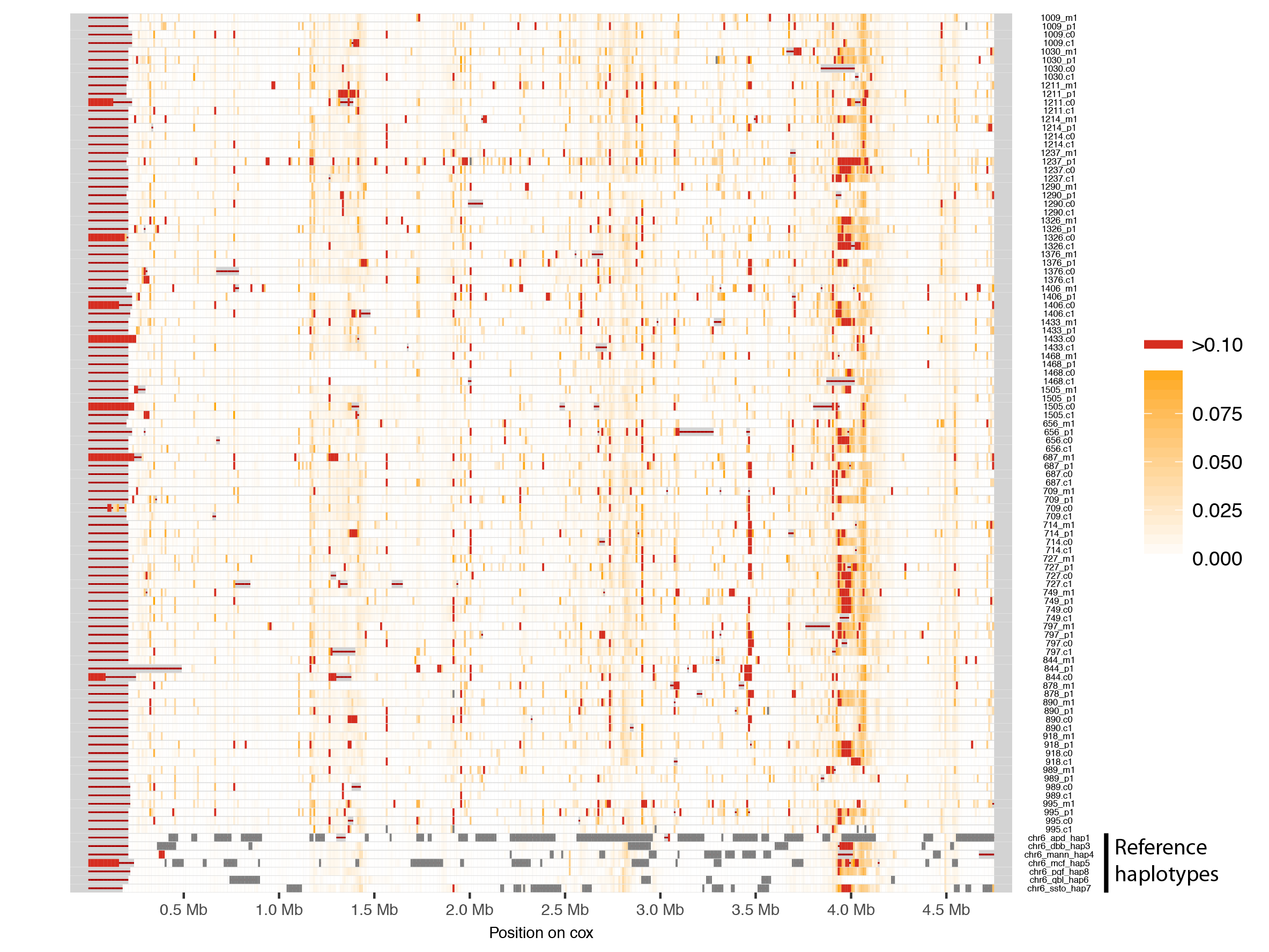
**

## Supplemental Figure S5. Differences between MHC haplotypes and reference cox.

The new haplotypes, the six alternative reference haplotypes and the pgf reference haplotype were aligned to the alternative reference haplotype cox through pairwise alignment and the percentage of pairwise differences was calculated in bins of 10kb (shown here in white (low) to red (high). Dark gray bins contain more than 50 % missing data (i.e. N’s), bins with red line lack alignment blocks.

## Supplemental Figure S6. Global MHC diversity.

Five diploid MHC regions were sampled from the 26 populations in the 1000 genomes project and compared with our new haplotypes using PCA (left panel) and a neighbor-joining tree (right panel).

## Supplemental Figure S7. Genotype concordance.

(a) Individual genotype concordance rates were calculated from all sites (n=2475) genotyped in all individuals in our call set by BayesTyper and The HumanCoreExome BeadChip v.1.0 used to genotype the same individuals. (b) The concordance rate calculated for all individuals across the MHC region.

**
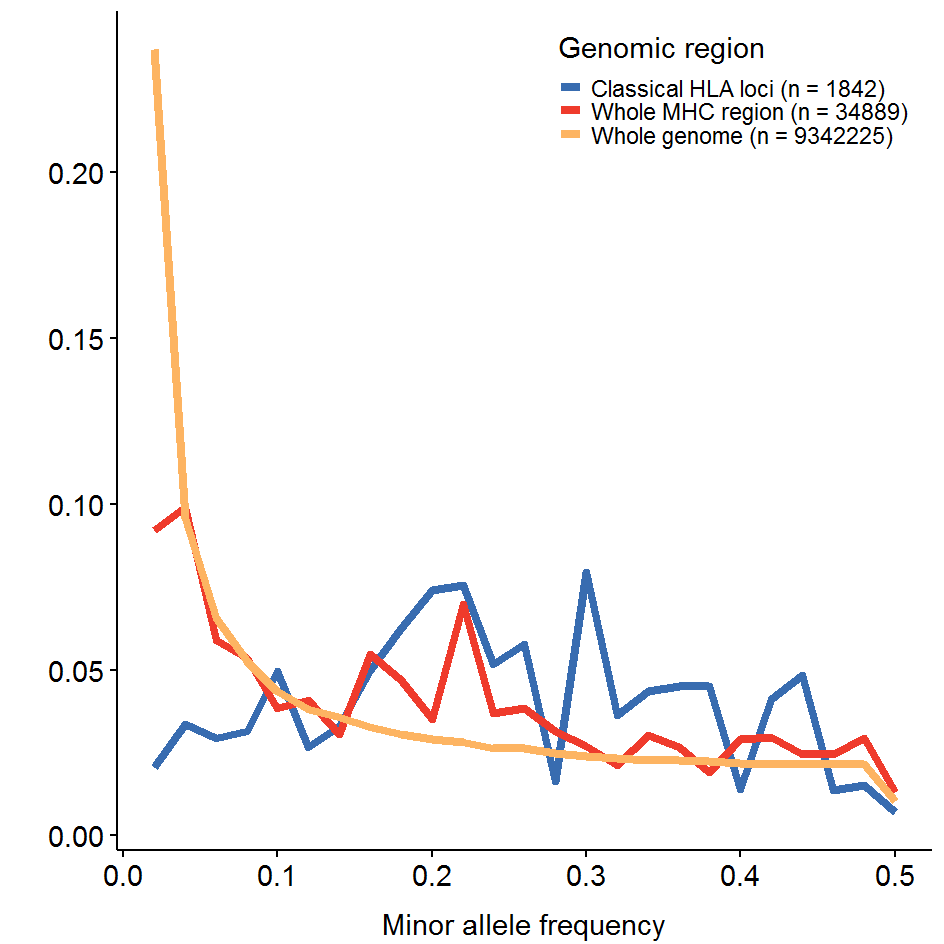
**

## Supplemental Figure S8. Folded site frequency spectrum.

The folded site frequency spectrum calculated for nine classical HLA genes (*HLA-A*, *HLA-C*, *HLA-B*, *HLA-DRA*, *HLA-DRB1*, *HLA-DQA1*, *HLA-DQB1*, *HLA-DPA1*, *HLA-DPB1*, blue), the whole MHC region (red) and the whole genome (yellow).

**
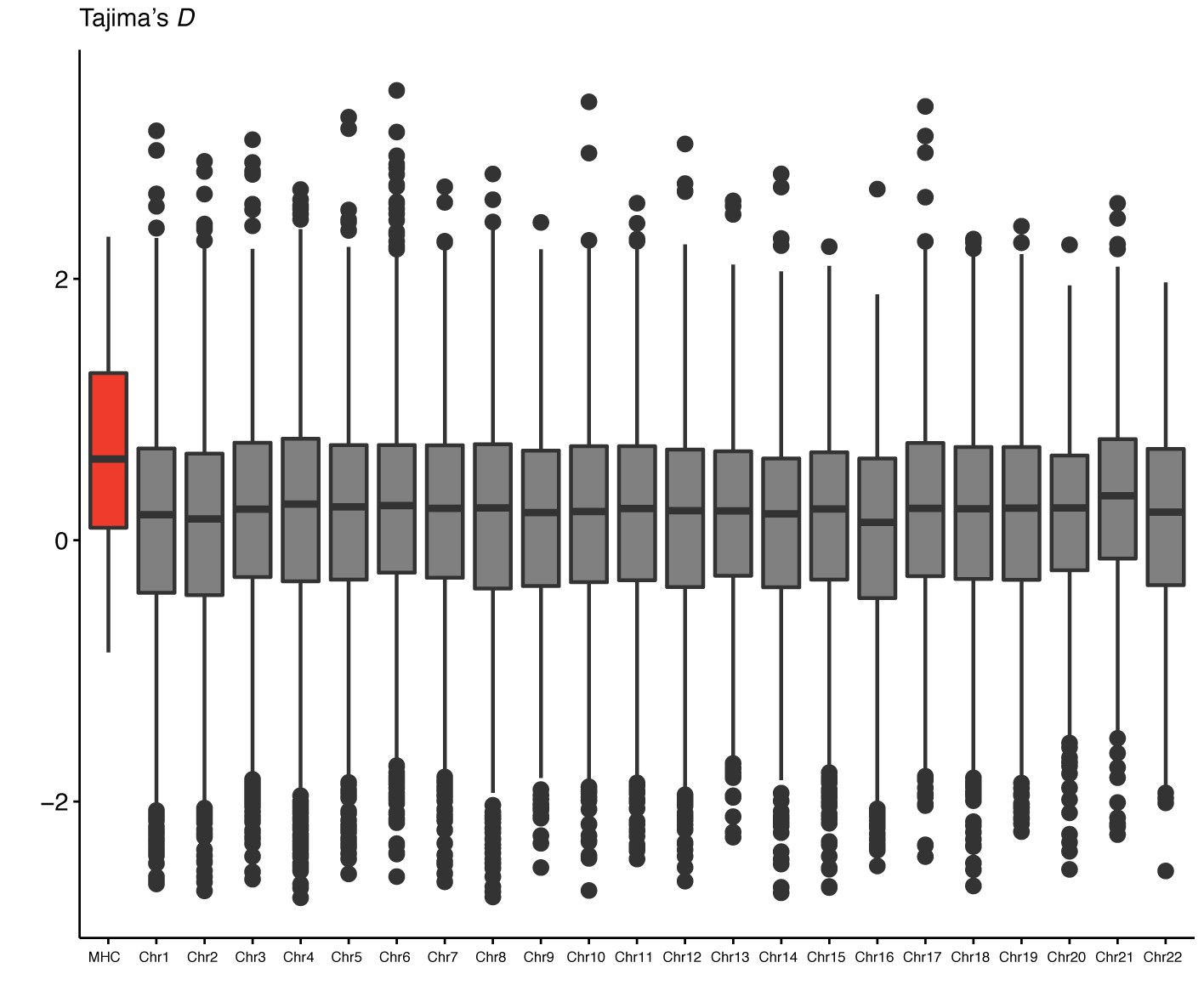
**

## Supplemental Figure S9. Genome wide Tajima’s *D*.

Tajima’s *D* statistic was calculated in bins of 100kb across the entire genome and the MHC-region and shown here as a boxplot for the MHC region and each chromosome.


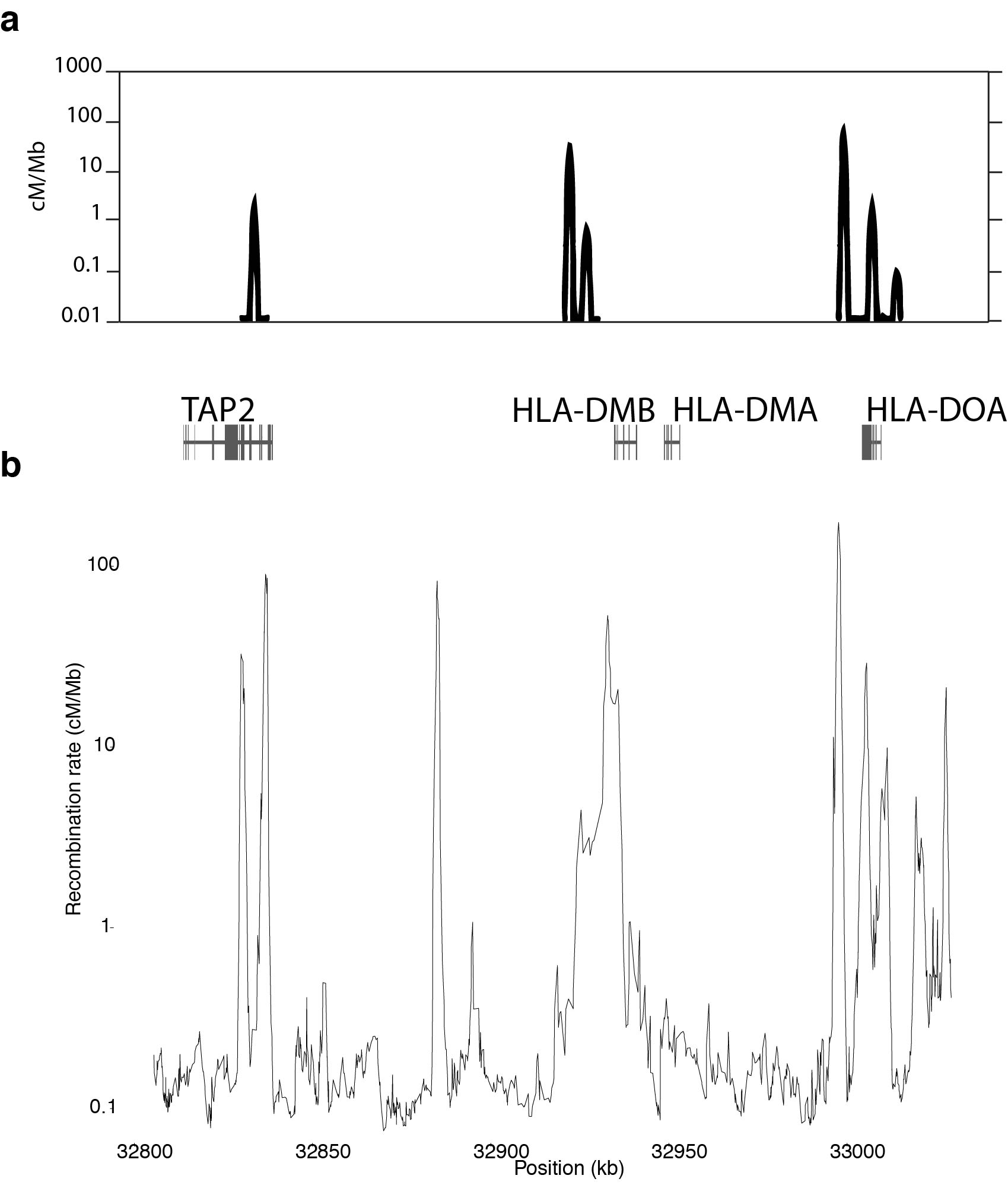


## Supplemental Figure S10. Recombination rate in 200kb region.

(a) Illustration of the finding of Jeffreys et al. (Jeffreys, A J, L Kauppi, and R Neumann. 2001.)*.* (b) Recombination rate from this study is shown for the same 200kb region.

**
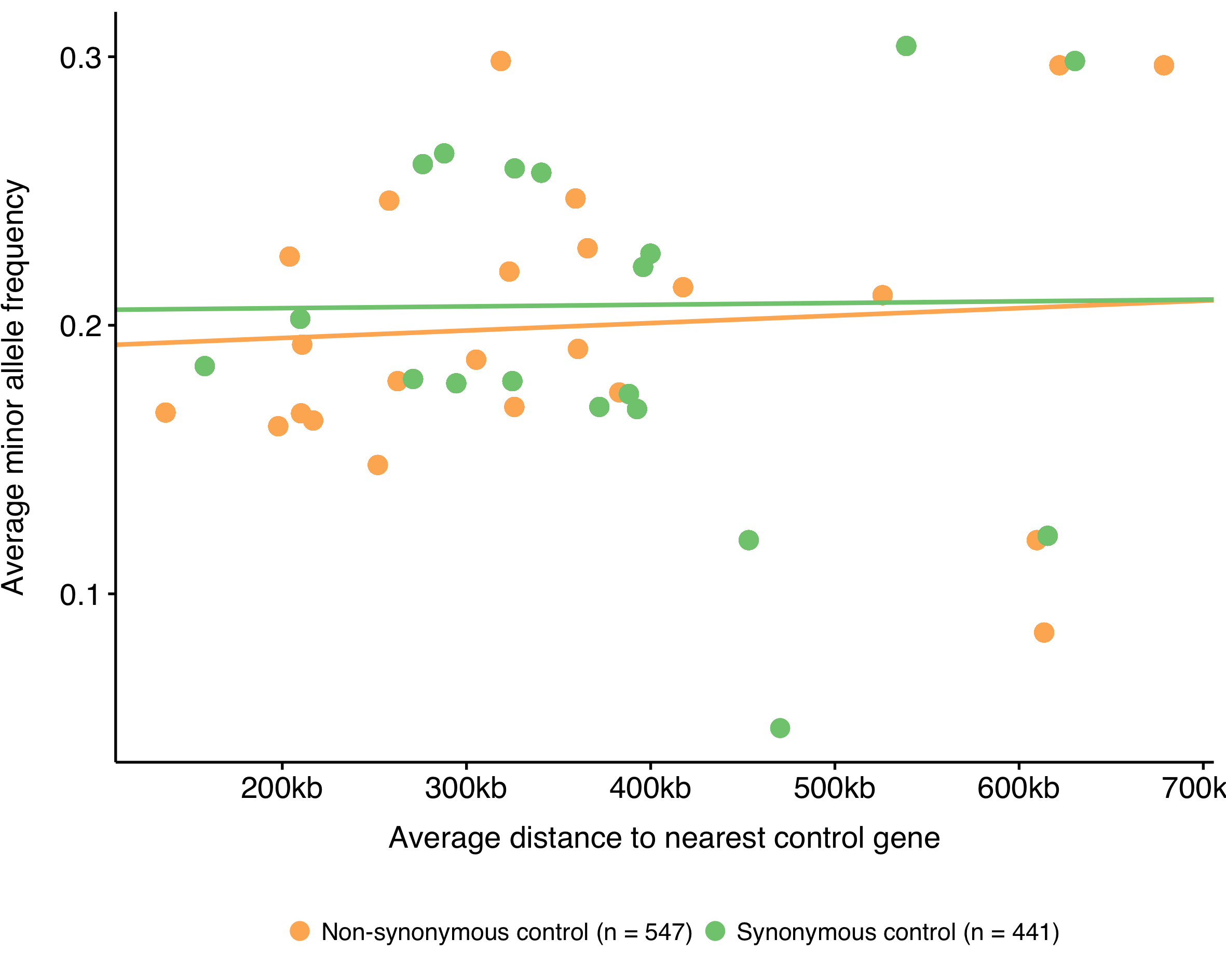
**

## Supplemental Figure S11. Linked selection control.

(Left panel) Nine control genes were chosen at random from all genes in the MHC region. Average minor allele frequencies of non-synonymous (yellow, n= 547, p-value < 0.05, notice however, that the slope is positive) and synonymous variants (green, n=441, p-value = 0.169) were calculated in bins of 25 variant sites and plotted as a function of the average distance of those 25 variants to the nearest control gene. Variants within the control MHC genes are not included. A linear regression was fitted for each variant type on the non-binned data.

# Supplemental Tables

## Supplemental Table S1. Validation.

The columns from left to right contain information on the haplotype-id, positions in the sequence of the haplotype (separated by a semicolon) that contain the variant, the number of variants, the fraction of those variants that were validated, the total length of the amplified region that was sequenced and the length of the region containing the variants, PCR fragment ID, the variant alleles, the expected phase of the variant alleles (variants connected by a dash are expected to be in the same haplotype), the actual phase observed, the number of correctly phased variants, missing data of allele or different allele than expected (indicated by an asterisk) and total number of variants that could be determined. The total number of variants and the overall validation rate is shown at the top.

# The Danish Pan Genome Consortium

Lasse Maretty^1^, Jacob Malte Jensen^2,3^, Bent Petersen^4^, Jonas Andreas Sibbesen^1^, Siyang Liu^1,5^, Palle Villesen^2,3,6^, Laurits Skov^2,3^, Kirstine Belling^4^, Christian Theil Have^7^, Jose M. G. Izarzugaza^4^, Marie Grosjean^4^, Jette Bork-Jensen^7^, Jakob Grove^3,8,9^, Thomas D. Als^3,8,9^, Shujia Huang^10,11^**,**Yuqi Chang^10^,  Ruiqi Xu^5^,  Weijian Ye^5^, Junhua Rao^5^**,** Xiaosen Guo^10,12^**,** Jihua Sun^5,7^**,** Hongzhi Cao^10^, Chen Ye^10^, Johan v Beusekom^4^**,** Thomas Espeseth^13,14^**,** Esben Flindt^12^, Rune M Friborg^2,3^, Anders E. Halager^2,3^, Stephanie Le Hellard^14,15^, Christina M Hultman^16^, Francesco Lescai^3,8,9^, Shengting Li^3,8,9^, Ole Lund^4^, Peter Løngren^4^, Thomas Mailund^2,3^, Maria Luisa Matey-Hernandez^4^, Ole Mors^3,6,9^, Christian NS Pedersen^2,3^, Thomas Sicheritz-Pontén^4^, Patrick Sullivan^16,17^, Ali Syed^4^, David Westergaard^4^, Rachita Yadav^4^, Ning Li^5^**,** Xun Xu^10^**,** Torben Hansen^7^, Anders Krogh^1^, Lars Bolund^8,10^, Thorkild IA Sørensen^7,18,19^, Oluf Pedersen^7^, Ramneek Gupta^4^, Simon Rasmussen^4^, Søren Besenbacher^2,6^, Anders D. Børglum^3,8,9^, Jun Wang^3,10,12^, Hans Eiberg^20^, Karsten Kristiansen^10,12^, Søren Brunak^4,21^, Mikkel Heide Schierup^2,3,22^

**Affiliations**

^1^Bioinformatics Centre, Department of Biology, University of Copenhagen, 2200 Copenhagen N, Denmark

^2^Bioinformatics Research Centre, Aarhus University, 8000 Aarhus C, Denmark

^3^iSEQ, Centre for Integrative Sequencing, Aarhus University, 8000 Aarhus C, Denmark

^4^DTU Bioinformatics, Department of Bio and Health Informatics, Technical University of Denmark, Kemitorvet, 2800 Kongens Lyngby, Denmark

^5^BGI-Europe, Ole Maaløes Vej 3, 2200 Copenhagen N, Denmark

^6^Department of Clinical Medicine, Aarhus University, 8000 Aarhus C, Denmark

^7^Novo Nordisk Foundation Center for Basic Metabolic Research, Section of Metabolic Genetics, University of Copenhagen, 2100 Copenhagen Ø, Denmark

^8^Department of Biomedicine, Aarhus University, 8000 Aarhus C, Denmark

^9^The Lundbeck Foundation Initiative for Integrative Psychiatric Research, iPSYCH, Denmark

^10^BGI-Shenzhen, Shenzhen 518083, China

^11^School of Bioscience and Biotechnology, South China University of Technology, Guangzhou 510006, China

^12^Laboratory of Genomics and Molecular Biomedicine, Department of Biology, University of Copenhagen, 2100 Copenhagen Ø, Denmark

^13^Department of Psychology, University of Oslo, Norway

^14^NORMENT, KG Jebsen Centre for Psychosis Research, Department of Clinical Science, University of Bergen, Bergen, 5021, Norway

^15^Dr E. Martens Research Group of Biological Psychiatry, Center for Medical Genetics and Molecular Medicine, Haukeland University Hospital, Bergen, 5021, Norway

^16^Department of Medical Epidemiology and Biostatistics, Karolinska Institutet, Stockholm, 17177, Sweden

^17^Department of Genetics, University of North Carolina, Chapel Hill, NC 27599-7264, USA

^18^Department of Clinical Epidemiology (formerly Institute of Preventive Medicine), Bispebjerg and Frederiksberg Hospital, The Capital Region, Copenhagen, Denmark

^19^Department of Public Health, Faculty of Health and Medical Sciences, University of Copenhagen, Copenhagen, Denmark

^20^Department of Cellular and Molecular Medicine, University of Copenhagen, 2200 Copenhagen N, Denmark

^21^Novo Nordisk Foundation Center for Protein Research, Faculty of Health and Medical Sciences, University of Copenhagen, 2200 Copenhagen N, Denmark

^22^Department of Bioscience, Aarhus University, 8000 Aarhus C, Denmark
